# Risk adapted diagnostics and hospitalization following mild traumatic brain injury

**DOI:** 10.1007/s00402-020-03545-w

**Published:** 2020-07-23

**Authors:** Lukas Leitner, Jasmin Helena El-Shabrawi, Gerhard Bratschitsch, Nicolas Eibinger, Sebastian Klim, Andreas Leithner, Paul Puchwein

**Affiliations:** Department of Orthopedics and Trauma, Medical University of Graz, Auenbruggerplatz 5, 8036 Graz, Austria

**Keywords:** TBI, Risk estimation, Intracranial bleeding, Prognosis, Hospitalization

## Abstract

**Introduction:**

Traumatic brain injury (TBI) remains a leading cause of hospital admission and mortality, intracranial hemorrhage (ICH) presents a severe complication. Low complication tolerance in developed countries and risk uncertainty, often cause excessive observation, diagnostics and hospitalization, considered unnecessary and expensive. Risk factors predicting ICH, progression and death in patients hospitalized with mild TBI have not been identified yet.

**Methods:**

Mild TBI cases indicated for cranial computer tomography (CT) and hospitalization, according to international guidelines, at our Level I Trauma Center between 2008 and 2018 were retrospectively included. Multivariate logistic regression was performed for ICH, progression and mortality predictors.

**Results:**

1788 mild TBI adults (female: 44.3%; age at trauma: 58.0 ± 22.7), were included. Skull fracture was diagnosed in 13.8%, ICH in 46.9%, ICH progression in 10.6%. In patients < 35 years with mild TBI, chronic alcohol consumption (*p* = 0.004) and skull fracture (*p* < 0.001) were significant ICH risk factors, whilst in patients between 35 and 65 years, chronic alcohol consumption (*p* < 0.001) and skull fracture (*p* < 0.001) revealed as significant ICH predictors. In patients with mild TBI > 65 years, age (*p* = 0.009), anticoagulation (*p* = 0.007) and neurocranial fracture (*p* < 0.001) were significant, independent risk factors for ICH, whilst increased age (*p* = 0.01) was a risk factor for mortality following ICH in mild TBI. Late-onset ICH only occurred in mild TBI cases with at least two of these risk factors: age > 65, anticoagulation, neurocranial fracture. Overall hospitalization could have been reduced by 15.8% via newly identified low-risk cases.

**Conclusions:**

Age, skull fracture and chronic alcohol abuse require vigilant observation. Repeated CT in initially ICH negative cases should only be considered in newly identified high-risk patients. Non-ICH cases aged < 65 years do not gain safety from observation or hospitalization. Recommendations from our data might, without impact on patient safety, reduce costs by unnecessary hospitalization and diagnostics.

## Background

Traumatic brain injury (TBI) remains a leading cause of hospital admission and mortality amongst trauma patients [1]. According to the Centers for Disease Control and Prevention (CDC), in the United States (US), more than 3 Mio. people experience TBI per year, resulting in approximately 2.5 Mio. emergency department visits (5% of emergency visits), of which 280.000 demand hospitalization, causing over 52,000 deaths [2, 3]. The economic impact of TBI was calculated as approximately $76.5 billion (in 2010 US dollar) per year, 90% of which is caused by cases demanding hospitalization [4].

Intracranial hemorrhage (ICH; subdivided into epidural hematoma (EDH), subdural hematoma (SDH), subarachnoid bleeding (SAB) and intracerebral bleeding) can occur in mild TBI cases and can lead to severe complications. Described risk factors for ICH are the presence of symptoms associated with mild TBI (severe headache, vomiting, seizure, amnesia, consciousness, focal neurological signs), signs of moderate and severe TBI, increased age, alcohol or other substance intoxication, anticoagulation therapy and signs of skull fracture and/or rhinoliquorrhea [5–8]. Since the absence of any of these signs cannot fully close out the eventuality of ICH, the use of cranial computer tomography scans (CT) for the screening of even minor head injury cases, has widely become clinical routine [6, 9–11]. Such screening is expensive, calculations revealed that the reduction of only 10% of CT scans performed on mild TBI could save more than $20 Mio. per year [12].

Consequently, the clinical decision on the extent of mild TBI screening is often based on consensus agreements or department own protocols [5, 13], to reduce health care expenditures and still keep patient safety on a high level. Still, especially in developed countries, where (1) medicolegal climate shows zero tolerance for misdiagnosis and (2) ageing population on oral anticoagulants, potentially increasing ICH rate following TBI [14] is increasing, CT screening and hospitalization is often broadly justified by doubt or patients demand.

Once, mild TBI cases have been selected and CT screening was performed, evidence on risk factors for case development and clinical outcome is lacking. This uncertainty in risk estimation might lead to excessive observation, diagnostics and hospitalization. Earlier studies revealed that identification of ‘low-risk’ cases, suitable for alternative strategies to inpatient observation can, with similar clinical outcome, increase patients satisfaction and save costs [15].

This study was conducted to analyze the progression and mortality risk in patients who had already been selected for CT screening and hospitalization following TBI. Study aim was the analysis of representative subgroups, their risk factors predicting ICH, CT progression and mortality, to allow (1) improved risk estimation on hospitalized mild TBI patients and (2) reduction of unnecessary diagnostics and hospitalization in low-risk cases.

## Methods

### Ethics statement

This study was approved by the local Institutional Ethical Review Board (Reference number: EK-Nr. 29-534 ex 16/17) and performed in accordance with relevant regulations; informed consent for data analysis was obtained from participants during a hospital stay, if possible.

### Study population

All adult patients (older than 18 years at trauma) diagnosed with mild TBI which were indicated for CT screening and/or hospitalization between 2008 and 2018 at our Level I Trauma Center, were retrospectively included in this study. Case history and clinical follow up were retrieved from our and associated centers’ intern data systems using keyword identification of all written examination reports. Complete mortality data/dates were received from centralized insurance data.

Medical history, diagnosis of mild TBI (Classified according to Centers for Disease Control and Prevention (CDC) at our institution: Including Glasgow Coma Scale score (GCS, best available score in 24 h) 13–15, duration of altered mental state or loss of consciousness < 30 min, duration of post-traumatic amnesia < 1 day, and severity of the head injury; [16, 17]), number and result (fracture, bleeding, progression) of performed CT scans, demographic data, anticoagulation and other concomitant diseases were collected from all patients.

### Institutional protocol for CT screening of TBI

CT screening is in line with the guidelines of the German Society for Neurosurgery [18] which is mainly implementing findings from the ‘Canadian CT Head Rule’ and the ‘New Orleans Criteria’ [9, 11, 19]. In short form, CT scan was highly indicated in mild TBI ‘risk cases’ with amnesia, neurologic symptoms, seizure, clinical signs of skull fracture and/or rhinoliquorrhea, known or suspected anticoagulation in patients older than 60 years. CT scan was occasionally indicated in cases of doubt and unclear trauma mechanism, severe headache, substance intoxication or evidence for high energy trauma.

Repetitive CT scan was indicated in clinical worsening or missing clinical improvement on a case by case basis. In cases with diagnosed ICH, follow-up CT was performed as indicated by an interdisciplinary decision with neurosurgeons in line with existing guidelines [18].

### Institutional protocol for hospital observation

Indication for hospitalization of TBI cases was in line with the guidelines of the German Society for Neurosurgery [18]. In short form, hospital observation was absolutely indicated in cases demanding neurosurgical intervention, unconsciousness, posttraumatic changes in the CT scan and in patients classified as ‘risk cases’ as described in the institutional protocol for CT screening. Relative indications were cases of doubt, severe headache, nausea, substance intoxication and unclear domestic care.

### Statistical methods

SPSS Statistics 20 (IBM, Armonk, NY) was used for data analysis. Comparisons between groups were made using the chi-square test for categorical variables and the Student *t* test for continuous variables when normally distributed. Multivariate logistic regression analysis as earlier described by Hart et al.[20], including significant factors from univariate analysis and age, was performed to identify independent factors predicting ICH, progression and mortality. A *p* value < 0.05 was considered statistically significant.

## Results

1788 mild TBI patients (female: 44.3%; age: 58.0 ± 22.7, min. 18.3—max. 103.7 years old at trauma) where hospitalization was indicated according to international guidelines, were retrospectively included in this study. Initial CT scans were performed in 96.2% of these cases, in which a neurocranial fracture was diagnosed in 13.8%, ICH in 46.9%.

Repetitive CT scans were performed in 44.2% during a hospital stay, which led to a diagnosis of progredient bleeding in 28.3% of these cases. Any surgical intervention during hospitalization was necessary in 22.3% of all cases, in 6.0% neurosurgical intervention of the neurocranial fracture or bleeding was performed; Patients were hospitalized for 8.5 ± 10.5 days. 2.5% (*n* = 44) of all cases died within 30 days (this interval was considered correlated to their initial trauma), 22.9% of all cases died during the complete follow-up of 3.9 ± 2.6 years.

### Patients younger than 35 years

In this group (*n* = 364) mortality within 30 days was 0.0%. ICH was detected in 25.3% (*n* = 89) of all cases in this age group.

Amongst mild TBI cases younger than 35 years, chronic alcohol consumption (*p* < 0.001) and neurocranial fracture (*p* < 0.001) revealed significant risk factors for ICH in the initial CT scan (Table 1), which remained significant after age adjustment in a multivariate regression model. No significant risk factors could be detected for bleeding progression (*n* = 12) in the follow-up CT scan, neither for mortality within 30 days, which did not occur at all in mild TBI cases in this age group. Follow-up CT scans were performed in 3.4% of cases without initial detection of ICH, none of these CT scans revealed novel evidence of bleeding.

**Table 1:** Risk factors für ICH, ICH progression in a follow-up CT scan and death following mild TBI, divided by age groups

	< 35 years						35–65 years						> 65 years					
	ICH	*p*	Progression	*p*	Death	*p*	ICH	*p*	Progression	*p*	Death	*p*	ICH	*p*	Progression	*p*	Death	*p*
No of cases	89 of 351		12 of 64		0 of 364		255 of 614		47 of 252		1 of 628		507 of 786		132 of 488		43 of 796	
Risk factor																		
Sex (female)	30 of 129	0.654	3 of 24	0.429	n.n		80 of 204	0.412	9 of 83	**0.026**	n.n		291 of 428	**0.037**	72 of 280	0.441	22 of 434	0.872
Alcohol abuse	17 of 34	**< 00.001**	4 of 9	0.099	n.n		59 of 91	**< 00.001**	13 of 52	0.187	1 of 93	**0.016**	31 of 51	0.548	4 of 25	0.200	1 of 52	0.250
Renal insufficiency	0 of 2	0.409	n.n		n.n		11 of 17	**0.049**	0 of 11	0.104	n.n		156 of 214	**0.003**	47 of 152	0.195	17 of 215	0.057
Diabetes	1 of 4	0.990	0 of 1	0.479	n.n		18 of 38	0.451	3 of 15	0.890	n.n		110 of 161	0.256	27 of 103	0.830	8 of 161	0.786
CHD	0 of 1	0.560	n.n		n.n		17 of 36	0.475	3 of 16	0.992	n.n		143 od 221	0.965	41 of 146	0.737	11 of 222	0.725
Cardial arrhythmia	n.n		n.n		n.n		4 of 7	0.399	1 of 4	0.742	n.n		105 of 160	0.740	32 of 104	0.336	13 of 160	0.088
Previous stroke	n.n		n.n		n.n		4 of 10	0.918	1 of 6	0.900	n.n		73 of 95	**0.007**	20 of 67	0.516	5 of 96	0.921
Previous TIA	n.n		n.n		n.n		1 of 6	0.214	0 of 2	0.497	n.n		19 of 29	0.907	6 of 19	0.650	3 of 30	0.256
Drugs																		
Anticoagulation	0 of 2	0.409	n.n		n.n		34 of 68	0.133	6 of 37	0.681	n.n		350 of 503	**< 0.001**	98 of 345	0.295	31 of 504	0.220
Dual therapy	n.n		n.n		n.n		4 of 7	0.399	2 of 4	0.105	n.n		16 of 24	0.822	3 of 15	0.532	2 of 24	0.519
ASS	n.n		n.n		n.n		27 of 45	**0.009**	3 of 26	0.326	n.n		225 of 309	**< 0.001**	55 of 207	0.838	19 of 310	0.469
Phenprocoumon	0 of 1	0.560	n.n		n.n		4 of 9	0.858	2 of 7	0.494	n.n		66 of 94	0.218	20 of 70	0.757	8 of 93	0.146
Plavix	n.n		n.n		n.n		2 of 11	0.113	1 of 2	0.253	n.n		43 of 61	0.309	13 of 41	0.483	2 of 61	0.445
Xarelto	0 of 1	0.560	n.n		n.n		2 of 5	0.944	1 of 2	0.253	n.n		13 of 25	0.184	3 of 17	0.374	3 of 26	0.159
Cumarin	n.n		n.n		n.n		0 of 2	0.223	0 of 1	0.631	n.n		12 of 21	0.475	8 of 14	**0.010**	0 of 21	0.267
Pradaxa	n.n		n.n		n.n		1 of 1	0.235	1 of 1	**0.036**	n.n		4 of 9	0.206	0 of 5	0.171	0 of 9	0.471
Asasantin	n.n		n.n		n.n		1 of 1	0.235	0 of 1	0.631	n.n		2 of 3	0.937	n.n		0 of 3	0.678
Eliquis	n.n		n.n		n.n		n.n		n.n		n.n		1 of 3	0.258	2 of 3	0.121	1 of 3	**0.032**
Lixiana	n.n		n.n		n.n		n.n		n.n		n.n		0 of 1	.177	n.n		0 of 1	0.811
Brilique	n.n		n.n		n.n		1 of 1	0.235	0 of 1	0.631	n.n		n.n		n.n		n.n	
Trauma																		
Polytrauma	11 of 30	0.134	1 of 8	0.817	n.n		26 of 69	0.483	6 of 29	0.764	n.n		11 of 19	0.542	1 of 9	0.277	0 of 19	0.292
Skull fracture	27 of 35	**< 0.001**	6 of 23	0.419	n.n		90 of 111	**< 0.001**	24 of 88	**0.010**	n.n		22 of 82	**< 0.001**	35 of 87	**0.002**	4 of 105	0.469
ICH																		
EDH			2 of 7	0.680	n.n				3 of 20	0.807	n.n				3 of 24	0.215	1 of 24	0.951
SAB			7 of 34	0.484	n.n				30 of 142	0.252	1 of 177	0.116			89 of 271	**0.001**	19 of 313	0.325
SDH			4 of 18	0.216	n.n				21 of 80	**0.035**	n.n				75 of 212	**< 0.001**	17 of 240	0.098
Intracerebral			6 of 24	0.359	n.n				29 of 92	**< 0.001**	n.n				51 of 148	**0.025**	15 of 160	0.056

Bold value indicates for statistical significance (*p* <  0.05)

Drugs have been ordered according to the frequency of occurrence; ‘alcohol abuse’ and ‘renal insufficiency’ are used for the chronic condition

*ICH* intracranial hematoma, *p* *p* value, *EDH* epidural hematoma, *SAB* subarachnoid hematoma, *SDH* subdural hematoma

Mild TBI cases younger than 35 years without ICH stayed in the hospital for 4.3 ± 7.5 days after the initial trauma, whilst cases with ICH were hospitalized significantly longer (6.8 ± 6.6 days; *p* = 0.006).

### Patients aged between 35 and 65 years

In this group (*n* = 628) ICH was detected in 41.5% (*n* = 225) of all cases. ICH was significantly associated with chronic alcohol consumption (*p* < 0.001) renal insufficiency (*p* = 0.049), intake of ASS (*p* = 0.009) and fracture of the neurocranium (*p* < 0.001) (Table 1), of which chronic alcohol abuse (OR 1.22; *p* < 0.001) and neurocranial fracture (OR 1.46; *p* < 0.001) remained significant, independent factors in an age-adjusted multivariate logistic regression analysis. Male sex, neurocranial fracture, subdural and intracerebral bleeding revealed significant factors for bleeding progression in the follow-up CT scan (Table 1), amongst which intracerebral bleeding (OR 1.17; *p* = 0.001) remained an independent significant factor in an age-adjusted multivariate logistic regression analysis.

Mortality within 30 days from trauma was only observed in one single case following mild TBI in this age group, interestingly a case with chronic alcohol abuse.

In this age group, mild TBI cases without ICH were hospitalized 7.1 ± 10.5 days after the initial trauma, whilst ICH cases were hospitalized significantly longer (8.9 ± 8.9 days; *p* = 0.029).

### Patients older than 65 years

The age group older than 65 years with mild TBI can be considered the most relevant patient group since it represented the largest subgroup (*n* = 786; 44.5% of all included cases) and had the highest ICH rate amongst mild TBI cases (*n* = 507; 63.7%). In this group, significant risk factors for ICH in the initial CT were female sex (*p* = 0.037) renal insufficiency (*p* = 0.003), history of stroke (*p* = 0.007), anticoagulation therapy (*p* < 0.001), ASS intake (*p* < 0.001) and neurocranial fracture (*p* < 0.001) (Table 1). Multivariate logistic regression analysis, including all significant risk factors and age, revealed age (OR 1.01 per year; *p* = 0.009), anticoagulation (OR 1.1; *p* = 0.007) and neurocranial fracture (OR 1.2; *p* < 0.001) significant, independent factors for ICH in mild TBI cases aged over 65 years.

Progression in the follow-up CT scan was observed in 132 cases, significant risk factors were coumarin intake (*p* = 0.010), neurocranial fracture (*p* = 0.002), SAB (*p* = 0.001), SDH (*p* < 0.001) and intracerebral bleeding (*p* = 0.025) (Table 1). Multivariate logistic regression analysis, including all significant factors and age, revealed neurocranial fracture (OR 1.15; *p* = 0.005), SAB (OR 1.14; *p* < 0.001), SDH (OR 1.16; *p* < 0.001) and intracerebral bleeding (OR 1.09; *p* = 0.006) significant, independent risk factors for ICH progression in a CT follow-up.

Whilst mild TBI cases aged over 65 years revealed a 5.4% 30-day mortality following initial trauma (*n* = 43, age: 82.7 ± 9.3 years), it increases to 8.5% in patients with ICH. No statistically representative, significant risk factor could be identified for mortality in this group (Table 1). Multivariate logistic regression analysis, including all factors with a *p* value < 0.1 and age, revealed that patients age (OR 1.01 per year; *p* = 0.01) was the only determining, independent risk factor for mortality following ICH in mild TBI cases aged over 65 years within 30 days after trauma.

Most deceased cases were highly multimorbid, a concise causality of death was often not documented. From cases with complete documentation available, 63.6% deceased causally related to TBI, the others most probably from further health state deterioration related to TBI or the initial trauma (e.g., pneumonia, probably aspiration induced).

In this age group, mild TBI cases without ICH were hospitalized 6.2 ± 8.2 days after theee initial trauma, whilst cases with ICH were hospitalized significantly longer (7.9 ± 6.2 days; *p* = 0.002).

### Risk group identification for mild TBI

**Fig. 1:**
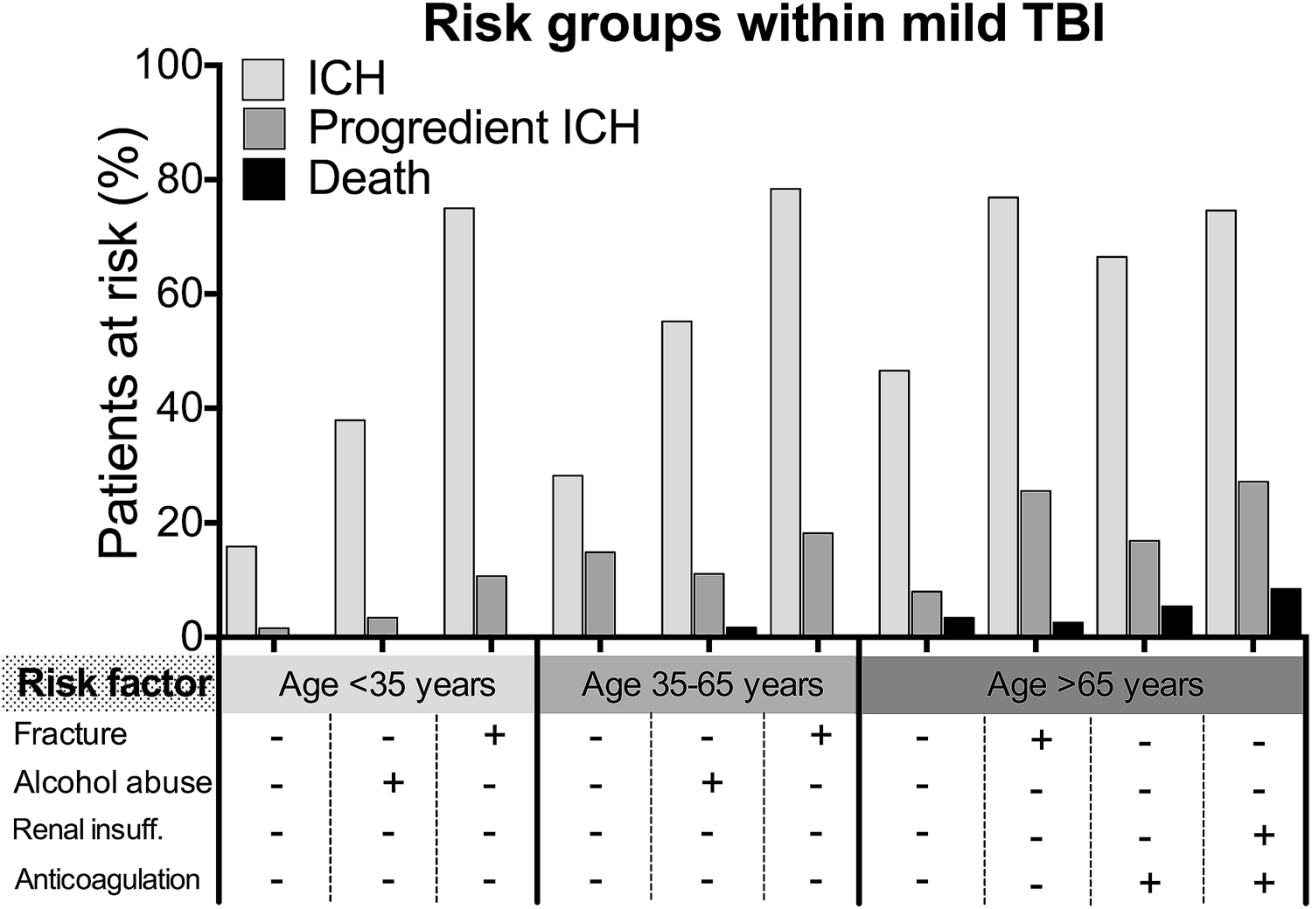
Graphical representation of selective risk groups amongst mild TBI cases, concerning ICH, progression and mortality risk; Graphs show that increasing age group tendentially leads to worsening of these parameters, further impaired by selected risk factors

Analysis of risk factors led to the identification of sub groups with increased risk of ICH, the progression of bleeding in the follow-up CT and death within 30 days following trauma (Fig. 1): Whilst patients younger than 35 years, who were admitted to hospital with mild TBI and no further risk factors, revealed a bleeding risk of 15.9%, a bleeding progression risk in follow-up CT of 1.6% and 0.0% mortality risk in our study population, presence of a neurocranial fracture in the CT increased the progression risk to 10.7%. Population aged > 65 had a much higher bleeding risk of 46.6% if no other risk factors were present, a bleeding progression risk of 8.0% and a mortality risk of 3.4%. When anticoagulation therapy and renal insufficiency were present at the same time in age > 65 years cases, bleeding risk was 74.6%, progression risk 27.2% and mortality risk within 30 days 8.5%. (Fig. 1).

### ICH in initially negative CT cases

Follow-up CT was performed in 110 mild TBI cases, where initial CT revealed no ICH, mainly due to the severity of symptoms; in this group 55 cases (50%) were older than 65 years. Interestingly follow-up CT revealed novel ICH in four cases (3.6%), all older than 65 years (80 ± 6.7 years) and all on anticoagulation therapy (2 cumarin, 1 phenprocoumon, 1 ASS), neurocranial fracture had been diagnosed in 3 (75%) cases in the initial CT scan.

### Potential cost reduction by reduced hospitalization

Mild TBI cases, younger 65 years, without ICH, without a history of alcohol abuse, who did not undergo any surgical intervention during their hospital stay, were selected (*n* = 462). In summary, these ‘low risk’ cases stayed in theee hospital for 1254 days (15.8% of overall 7945 days) for observation which cost approximately 1.2 Mio USD (considering 942 USD/day in the hospital as calculated by Austrian Health Authorities in 2017).

## Discussion

Patients admitted to hospital with mild TBI still present a high factor of uncertainty, concerning further risk-adapted diagnostics and observation. Especially in elderly patients, risk factors for ICH in mild TBI cases, have been largely investigated [5–8].

All patients admitted to our Level I Trauma Center with TBI within a decade (2008–2018) were screened according to international guidelines on CT screening and hospitalization [18]. Triggered by decreasing diagnostic error tolerance, international guidelines on CT screening and hospitalization of these patients, focus on safety maximization.

Known risk factors for complications in mild TBI cases, namely age > 65, chronic kidney disease, anticoagulation therapy, chronic alcohol abuse and neurocranial fracture, have earlier been identified [5–8, 21–23], and were in line with our results, supporting the comparability of our population to earlier published data. One exception was, that our mild TBI cases had a higher ICH rate compared to internationally published data [8, 19, 24]. An explanation could be, that clinically severe cases and cases with diagnosed ICH were transmitted to our Level I Trauma Center by local centers, negatively screened cases were vice versa sent to lower-level units.

Earlier publications suggest a correlation between skull fracture and ICH [25, 26], which is in line with our findings. Our data adds evidence, that skull fracture is a significant, independent risk factor for ICH formation in mild TBI cases in all age groups, and progression in patients aged > 65 years. The highly significant correlation between skull fracture and ICH in patients aged < 35 years (Fig. 1) is an indicator of highly energetic underlying trauma in these young patients, as we suppose.

**Fig. 2:**
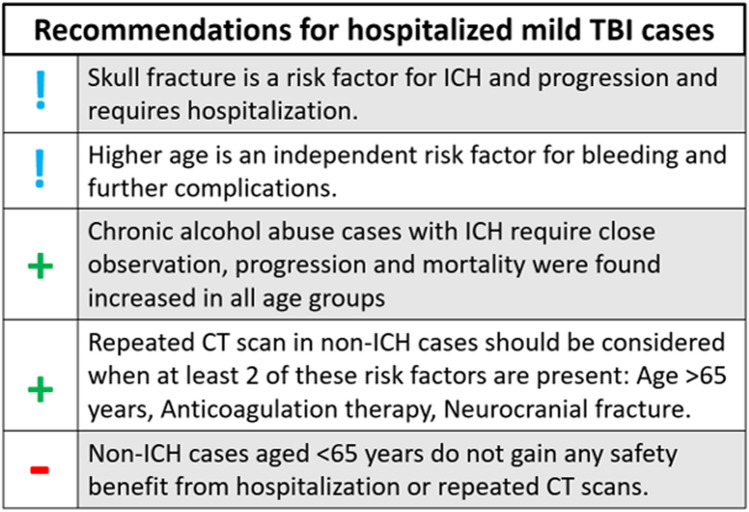
Recommendations that can be drawn from our analysis of TBI patients within a decade. (**!**), factors requiring vigilance; (**+**), positive recommendation; (−), negative recommendation;

Whilst broad evidence and international guidelines support a clinical decision on screening and hospitalization, further management of patients hospitalized for mild TBI remained unclear so far, since concrete risk factors, requiring vigilance were unknown in detail [3, 26, 27]. We hypothesized, that this lack of knowledge, via risk avoidance, leads to excessive diagnostics and unnecessary length of hospitalization in ‘low risk cases’—which can actually be concluded from our data. We further aimed to provide detailed knowledge on progression and mortality factors in mild TBI patients (Fig. 1), allowing better, risk-adapted case handling (Fig. 2), as our data suggest:

Patients with mild TBI and initial CT without detection of bleeding, but ongoing or progressive TBI symptoms, often trigger clinicians to perform a follow-up CT scan during their hospital stay—which was the case in 12.4% of our patients. During 10 years of observation, none of these scans led to the detection of newly occurred bleeding in mild TBI cases younger than 65 years (making up 50% of these cases). Our data suggest consideration of follow-up CT, in initially negative mild TBI cases, only if at least two of these risk factors are present: age > 65, anticoagulation therapy, neurocranial fracture.

Within 10 years of observation, none out of 621 mild TBI patients aged < 65 years, revealed late-onset ICH during their further course, no case died within 30 days. From all 965 mild TBI cases in this age group, including primary ICH cases, we could only detect one case of mortality (following ICH/SAB progression), who was an alcohol addict. Similar case reports on chronic alcohol abuse, ICH progression and mortality following TBI, have earlier been discussed in the literature [27, 28]. In these cases, even if aged < 65 years, we suggest higher vigilance.

Follow-up CT is suggested in all ICH cases following TBI since progression can never be fully ruled out in any risk constellation [26], which is supported by our data. Low tolerance for undetected complications in ICH cases also supports this procedure in all cases, where progression has a clinical implication. On the other hand, according to our data, non-elderly patients without ICH, repetitive CT or hospitalization, only cause costs without safety benefit.

Strikingly, our calculations revealed that non-hospitalization of non-ICH mild TBI cases < 65 years, cases with hospital stays for surgical interventions for additional injuries excluded, would have reduced the absolute number of TBI associated hospitalization days by 15.8%, saving scarce resources (lack in hospitalization slots) and 1.1 Mio. USD within the observation period.

These resources could be spent more effectively on rehabilitation and prevention programs, recent publications found increased depression, alcohol use disorder and gambling rates in patients following TBI [29–31].

### Limitations must be considered when our data is interpreted:

Cases were retrospectively included, with a TBI severity selection bias of a Level I Trauma Center. This could result in underreporting of ‘low-risk cases’, and consequently have reduced the calculated cost reduction by our suggestions, since these cases were transmitted to external units.

Comparison of hospitalization length between subgroups might exclude adjacent stays in external institutions, often administered following acute treatment at our institution. This might explain shorter mean hospitalization in elderly patients, for whom the availability of such external institution is better.

Furthermore, concomitant injuries to TBI, which might also have demanded extended hospitalization, have not been completely considered in all included 1788 cases—since they might be equally contributed between all groups.

## Conclusion

Evidence, retrieved from mild TBI cases treated at our Level I Trauma Center within a decade, allowed the identification of risk factors correlated with complications and mortality, enabling more risk-adjusted hospitalization and diagnostics.

We believe that recommendations based on our data might reduce costs by unnecessary hospitalization and diagnostics, without negative impact on patient safety.
